# Adoptive Cell Therapy: A Novel and Potential Immunotherapy for Glioblastoma

**DOI:** 10.3389/fonc.2020.00059

**Published:** 2020-01-31

**Authors:** Jingyu Wang, Fang Shen, Ying Yao, Lin-lin Wang, Yongjian Zhu, Jue Hu

**Affiliations:** ^1^School of Basic Medical Sciences and Forensic Medicine, Hangzhou Medical College, Hangzhou, China; ^2^Department of Neurosurgery, Second Affiliated Hospital, Zhejiang University School of Medicine, Hangzhou, China; ^3^Department of Orthopaedic Surgery's Spine Division, The Affiliated Hospital of Medical School of Ningbo University, Ningbo, China; ^4^Department of Basic Medicine Sciences, School of Medicine, Zhejiang University, Hangzhou, China

**Keywords:** adoptive cell therapy, Glioblastoma multiforme, chimeric antigen receptor, T cell receptor, tumor-infiltrating lymphocyte

## Abstract

Glioblastoma multiforme (GBM) is the most common primary brain tumor in adults with very poor prognosis and few advances in its treatment. Recently, fast-growing cancer immunotherapy provides a glimmer of hope for GBM treatment. Adoptive cell therapy (ACT) aims at infusing immune cells with direct anti-tumor activity, including tumor-infiltrating lymphocyte (TIL) transfer and genetically engineered T cells transfer. For example, complete regressions in patients with melanoma and refractory lymphoma have been shown by using naturally tumor-reactive T cells and genetically engineered T cells expressing the chimeric anti-CD19 receptor, respectively. Recently, the administration of ACT showed therapeutic potentials for GBM treatment as well. In this review, we summarize the success of ACT in the treatment of cancer and provide approaches to overcome some challenges of ACT to allow its adoption for GBM treatment.

## Introduction

Gliomas, including astrocytoma, oligodendroglioma, mixed glioma, medulloblastoma, and ependymoma, are the most common primary central nervous system (CNS) tumors that arise from glial or its precursor cells (1). Glioblastoma multiforme (GBM), the highest grade (WHO IV) astrocytoma, is the most prevalent type in adults. It has been investigated that more than 11,000 individuals suffered from GBM each year in the United States. In the last 30 years, survival rates for patients with GBM have improved very little. Despite aggressive standard therapies (maximal safe surgical resection, radiation, and temozolomide), outcomes for patients with newly diagnosed GBM remain dismal. The median survival of GBM is fewer than 20 months and a 5-year survival rate is merely 4–5% (2–5). Moreover, treatments for GBM are among the costliest with the least return, bringing a significant burden to society.

Over the last decade, emerging immunotherapy aimed at improving specific immune response against tumor cells has brought a glimmer of hope to patients with GBM. Generally, immunotherapy can be divided into four aspects, including monoclonal antibodies (mAb) to the inhibitory immune checkpoint molecules, oncolytic virus therapy, adoptive cell therapy (ACT), and cellular vaccines therapy (6–9).

The immune inhibitory molecules such as cytotoxic T lymphocyte-4 (CTLA4) and programmed death 1 (PD-1) are expressed on the surfaces of T cells. When bounding by their ligands expressed on tumor cells or macrophages, these molecules inhibit T cell's activation and proliferation, resulting in tumor immune escape (10). Nowadays, anti-PD-1/PD-L1 therapy has become a routine treatment option for patients with tumors highly expressing PD-L1, such as lung cancer and melanoma. High expression of PD-L1 has also been identified in GBM, which accounts for approximately 50% of newly diagnosed GBM and 45% of recurrent GBM, respectively. Patients with PD-L1 expression are predicted to have a worse prognosis, suggesting anti-PD1/PDL-1 is a potential GBM therapy target (11, 12). However, in a phase 3 clinical trial (NCT02017717), patients with recurrent GBM received nivolumab (anti-PD1 immunotherapy) showed no notably difference in overall survival (OS) compared with another group who treated with bevacizumab (an anti-VEGF therapy) (13). It may be due to the relatively low mutant load, few T cells' infiltration, and severe immunosuppressive microenvironment in GBM. Additionally, exclusively using anti-PD-1/PDL-1 will cause the activation of other inhibitory signals such as T cell immunoglobulin mucin-domain containing-3 (Tim3), lymphocyte activation gene 3 (LAG3), and CTLA4, becoming another approach of immune escape (14). A combination of immune checkpoint inhibition has shown anti-tumor response and promoted survival in animal models with GBM, whereas more clinical trials are needed to prove the efficacy and safety of immune checkpoint inhibitors treatment (15, 16). Certainly, blood-brain barrier (BBB) obstructed antibodies entry into brain, which should be further resolved.

Oncolytic Viruses (OVs) are a group of viruses with the ability to specifically infecting tumor cells and inducing tumor lysis. Recent clinical trials revealed OVs therapy, including using recombinant adenovirus DNX-2401, polio-rhinovirus chimera, and parvovirus H-1, was able to prolong the survival of patients with GBM (>30 months of survival after treatment) (17). However, valid viral spread and replication can be resisted via cancer stem cells and innate immune cells that occur in the GBM microenvironment (18).

Tumor vaccines therapy is aimed at stimulating patients' immune systems to produce tumor-specific immune cells by transferring tumor-associated antigens. Dendritic cells (DCs) can be pulsed with a wide variety of tumor-specific antigen sources (synthetic peptides or autologous tumor lysate). After binding with MHC molecules, these antigens can be presented on DCs' surfaced to stimulate the response of T cells. Injection of DCs-based vaccine into patients with GBM can induce intracranial T-cell infiltration and anti-tumor effects (19). A clinical trial revealed 41% of patients suffered from GBM exhibited cytokine responses and survived at least 2 years after injecting autologous DC pulsed with tumor lysate (20). Moreover, vaccines combined with an adjuvant such as toll-like receptor agonists can boost continuous immune responses (21).

Adoptive cell therapy (ACT), including tumor-infiltrates lymphocytes (TILs) transfer and genetically engineered T cells transfer, is one of the most significant breakthroughs in the field of immune-oncology. Chimeric antigen receptor (CAR) engineered autologous T cells have produced sustained remissions in refractory lymphomas, but it needs further study in the treatment of solid tumors (22–24). Adoptive transfer of mutation-reactive TILs has led to durable regression in cancer such as breast cancer, lung cancer, and melanoma (25–27). In this review, we will review and focus on GBM targeted ACT.

## Current Therapeutic Strategy for Glioblastoma

Over the last 15 years, the Stupp protocol, which is maximum safe tumor resection, followed by radiotherapy (RT) and temozolomide (TMZ) chemotherapy, has become the current standard first-line therapy for adult patients with newly diagnosed GBM. It has been shown that this regimen led to a median OS of 14.6 months in the combined therapy group (RT-TMZ) vs. 12.1 months in the RT only group. Methylation of the MGMT promoter was the predictor for a better outcome (28).

In addition, a new electric-physical cancer treatment modality (TTFields) was proved by the FDA in 2015 for GBM patients. A phase III trial has indicated that a better median OS occurs in GBM patients who received TTFields on the basis of standard therapy (29). However, the incremental cost-effectiveness ratio for these patients with tumor-treating fields (TTF) therapy is *e*549 909 per life-years gained, bringing a significant burden to the family (30). Unfortunately, the great majority of trials have revealed that more than 100 different molecularly targeted drugs showed little efficacy but increased risks of adverse events for newly diagnosed or recurrent GBM patients (31, 32). In view of the limited efficacy of standard therapy and a lack of effective molecularly targeted drugs, there is a strong interest in the development of personalized immunotherapy such as ACT.

## Adoptive Cell Therapy

### TIL Transfer

Adoptive cell therapy was initially pointed out by Rosenberg in 1982. He discovered that the administration of immune lymphocytes expanded in IL-2 was able to cure mice with subcutaneous lymphomas (33). Meanwhile, it has been demonstrated that the administration of a high dose of IL-2 after lymphocytes' transfer enhanced the therapeutic efficacy of ACT (34). In addition, the sustainable regression of established liver and lung tumors was mediated via TILs transfer (35). In melanoma, researches revealed that human TILs could recognize autologous tumors, and the administration of these TILs led to the regression of melanoma (36). A subsequent trial has shown that 21 of 43 patients underwent objective regressions of metastatic melanoma (37). Moreover, the regressions of brain metastases (13 of 17 patients) have also been observed, suggesting that TILs can pass the blood-brain barrier and infiltrate into brain tumors (38). In these trials, 22% of patients had complete regressions of melanoma and 20% of patients did not have recurrences 5–10 years after TILs transfer (25). In addition, 55% of patients from the NCI, 48% of patients from the MD Anderson Cancer Center, 38% of patients from the Moffitt Cancer Center, and 40% of patients from the Ella Cancer Institute have shown an objective response to TILs transfer (39–41).

Theoretically, whole-exome sequencing has confirmed that TILs amplified from melanoma tissues could recognize non-synonymous cancer gene mutation products. For example, 25-amino acid polypeptides containing mutated amino acid in the middle were established and presented on the antigen-presenting cell (APC) surface to identify the immunogenic mutations (42). But just little mutations were recognized by TILs, especially in gastrointestinal and breast cancers (27, 42, 43). A trail has identified and purified KRAS (G12D)-reactive T cells from TILs cultured from one patient with metastatic colorectal cancer. And objective regression of all lung metastases was observed after the infusion of these T cells, but progression occurred in a lesion 9 months after treatment, which was due to the absence of HLA-C^*^08:02 (26). Moreover, a recent trial has revealed complete durable regression of chemorefractory hormone receptor (HR)-positive metastatic breast cancer in a patient after adoptive transfer of mutant-specific TILs (reactive against SLC3A2, KIAA0368, CADPS2, and CTSB) combined with IL-2 and PD-1 treatment, which provides a new individualized immunotherapy approach for cancer (27).

There was a pilot study demonstrated that the delivery of autologous TILs and IL-2 was effective for GBM patients (44). In this research, six patients underwent surgery followed by infusion of TILs and IL-2 in conjunction with chemotherapy. Three of these patients were diagnosed with anaplastic astrocytoma, 1 of these patients had a complete regression after 45 months, and 2 had a partial regression. Of other patients diagnosed with GBM, 2 had a partial regression. Dillman's group demonstrated that GBM patients who treated with lymphokine-activated killer (LAK) cells had a better prognosis compared with contemporary GBM patients (45). Subsequently, a further clinical trial (NCT00331526) was performed to explore the effect of LAK for GBM. In this study, 33 patients were received LAK cells with surgery and radiation therapy. The median survival of these patients from diagnosis of GBM was 20.5 months (46). These results supported the potential of TIL immunotherapy in GBM. But the outcome of these trials was not optimistic, which may be due to the therapeutic cells that were amplified from PBMCs. Thus, there may be low ratios of therapeutic cells that can attack tumor. Identification and purification of tumor-reactive TILs may be the key to the therapeutic success of TILs transfer in GBM. Theoretically, TILs isolated from GBM specimens possess the characteristics of diversity, divergence, and exhaustion, which indicate GBM might be recognized by these TILs (47, 48). And further studies have demonstrated that TILs cultured from patients' GBM could react against tumor cells (49, 50). In addition, exhausted molecules appear to be highly expressed in amplified TILs, and immune checkpoint therapy may promote the antitumor efficacy of TIL transfer therapy (27).

**Figure 1:**
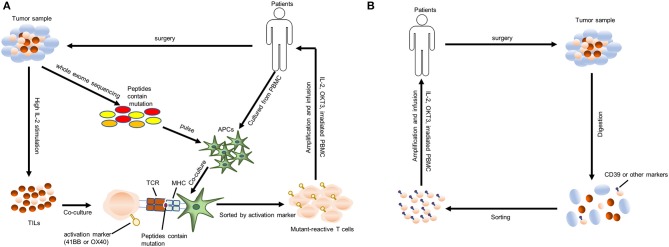
Protocols for identifying and transferring tumor-reactive T cells. **(A)** A “blueprint” for the therapy of patients using autologous T cells that recognize tumor-specific mutations. TILs, tumor-infiltrating lymphocytes; APCs, antigen-presenting cells; MHC, major histocompatibility complex; TCR, T cell receptor; PBMC, peripheral blood mononuclear cell. **(B)** A “blueprint” for the therapy of patients with autologous T cells recognizing tumor cells that were sorted by chronic activation markers such as CD39.

The latest protocol for transferring tumor-specific human TILs is shown in Figure 1A. Briefly, the resected tumor specimen is divided into ~1–2 mm fragments and then cultures individually in a high concentration of IL-2 (and IL-15, IL-21 if necessary). After 2-4 weeks of culture, lymphocytes overgrow, and are tested for reactivity against tumor-associated mutations. Then these selected TILs were sorted and rapidly expanded using irradiated allogenic PBMC and OKT3 antibody. After 1–2 weeks' amplification, up to 10^11^ TILs are collected for infusing into patients (51). lymphodepleting preparative regimen is necessary for patients before TILs transfer, which promotes cell persistence and durable immunoreaction. The general lymphodepleting preparative regimen includes the administration of 5 mg/m^2^ fludarabine for 5 days and 60 mg/kg cyclophosphamide for 2 days before cell infusion. After infusion, IL-2 is given at the dose of 720,000 IU/kg to induce tolerance (37). The administration of immune checkpoint agents may also promote cell persistence. However, the above protocol is time- and money-consuming, which needs a 2–3 months processing time after tumor resection with a low success rate. Some patients may die during the production of tumor-specific TILs. Therefore, simplification of processing protocol may be the research priority in the future. Recent researches have shown that CD39^+^ CD8^+^ T cells in the tumor microenvironment are tumor-reactive (52, 53). Therefore, we hypothesize that directly amplifying CD39^+^ CD8^+^ T cells sorted from solid tumors and infusing these T cells into patients may be a valid and more timesaving approach. The modified process is shown in Figure 1B, and, of course, it needs further study to test its efficacy and safety. Additionally, more molecular markers such as CD39 should be unveiled to facilitate the identification of tumor-reactive TILs.

### Genetically Engineered T Cells Transfer

#### CAR-T Cell Therapy

**Figure 2:**
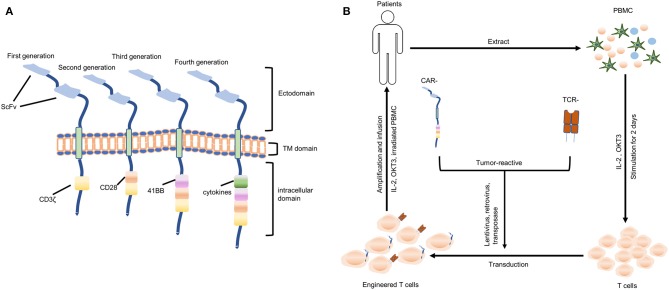
Genetically engineered T cells (CAR- and TCR-) transfer. **(A)** Structures of four generations of CAR-T cells. ScFv, single-chain variable fragment; TM, transmembrane. **(B)** A “blueprint” for the therapy of patients using autologous genetically engineered T cells (CAR T-cells or TCR T-cells).

CARs are generally composed of ectodomain (antigen recognition domain), transmembrane (TM) domain and an intracellular domain (signaling domain). And they are genetically expressed on the surface of T cells, allowing these T cells to directly recognize the tumor-associated antigen (TAA) independent of major histocompatibility complex (MHC) molecules. The ectodomain consists of a single-chain variable fragment (scFv) of a monoclonal antibody (MAb) that is specific for TAA or a ligand if the TAA is a cell surface receptor. The intracellular domain of first-generation CARs incorporates CD3ζ chain to mediate the activation of T cells. Second-generation CARs contain an additional intracellular domain of CD28, a co-stimulatory molecule. And third-generation CARs contain both CD28 and a tumor necrosis factor receptor family member such as CD 27, CD137 (4-1BB), ICOS, CD134 (OX40), or CD244, promoting the proliferation and persistence of T cells. More recently, fourth-generation CAR-T cells, also called “TRUCK” T cells, provided with stimulatory cytokines including IL-12, IL-15, IL-18 that antagonize the immunosuppressive tumor environment (Figure 2A) (54–58). Nowadays, CD28 combined with CD137 is the most commonly used co-stimulatory domain (59). CD28 signaling induces effector memory differentiation and CD137 signaling maintains a central memory phenotype. However, there is no direct clinical evidence supporting the superiority of CD28 combined with CD137 over other combinations.

Clinical trials involved administration of anti-CD19 CAR gene therapy in humans have achieved unprecedented success in refractory lymphoma. Infusion of anti-CD19 autologous CAR T-cells produced complete regression in patients with refractory lymphoma who maintains progression-free 4 years after two cycles of therapy (60). Anti-CD19 CAR T-cell therapy can lead to B cell exhaustion, which can be overcome via the periodic immunoglobulin infusions. However, solid tumors typically lack shared or specific surface antigens and therefore, there is the limited application of CAR T-cell therapy in them.

Nevertheless, there have been multiple trials of CAR T-cell therapy for GBM. Human epidermal growth factor receptor 2 (HER2) is overexpressed in some GBM. And a phase I trial (NCT01109095) has revealed that 17 patients with progressive GBM have no severe adverse events after the administration of autologous HER2-CAR T cells. And 8 of which patients had a clinical benefit with a median OS of 24.5 months (61). Moreover, it has been shown that there is an epidermal growth factor receptor (EGFR) overexpression in GBM. And EGFR variant 3 (EGFRvIII) is a frequently occurring mutation in GBM (62). Administration of CAR-T cells targeting EGFRvIII led to regression of GBM in the animal model, while it has been failed in a pilot trial (NCT01454596) (63–65). The loss of EGFRvIII in recurrent tumors may account for this failure, and EGFRvIII may not be the driven mutation in GBM (64). In addition, a recent clinical trial (NCT02208362) showed that the regression of all intracranial and spinal lesions was observed in a patient with recurrent multifocal GBM who received CAR-T cells targeting the interleukin-13 receptor alpha 2 (IL13Rα2) (66). The clinical response continued for 7.5 months until tumors eventually recurred at four new locations in the spinal cord, and there was low IL13Rα2 expression in these new lesions. Above trials have demonstrated the limited clinical benefits obtained from single-epitope CAR-T cell therapy. Multivalent CAR T-cells may overcome the problem of antigenic variability and immune escape of GBM. Recently, a trivalent CAR T-cell targeting 3 targetable glioma antigens (IL13Rα2, HER2, and ephrin-A2 [EphA2]) was designed, which could recognize almost 100% GBM (67). Moreover, podoplanin (PDPN), CD70, and IL7 Receptor are emerging targets of CAR T-cell therapy for GBM (68–70). In diffuse intrinsic pontine glioma (DIPG) with mutated histone H3 K27M (H3-K27M), disialoganglioside 2 (GD2) is highly expressed, and GD2-targeted CAR T-cells have shown a robust generation of antigen-dependent cytokine, killing of DIPG cells *in vitro*, and complete regression of DIPG in a mouse model (71). Driven antigens or receptors that are not shared with essential organ/tissue cells are required to be discovered in GBM.

#### TCR-T Cell Therapy

The T cell receptor (TCR) expressed on the surface of T cells recognizes antigenic peptides with MHC restriction. TCR heterodimer contains two-chains, either αβ or γδ, and αβ TCR is the most commonly used molecule for genetic engineering. The cDNAs for the TCR α and β chains can be sequenced and molecularly cloned from tumor-reactive T cells. The genetic transfer of this TCR is able to produce tumor antigen-specific T cells from autologous T cells. However, direct transfer of α- and β- chains can result in mispairing between endogenous and transgenic TCR-derived α- and β- chains. The mispairment of TCR will not recognize tumor antigens and may even target normal cells. Multiple strategies have been developed to resolve this problem, including replacing human regions with murine C regions, application of small interfering RNA constructs that inhibit the expression of endogenous TCR, TCR chain leucine zipper fusions, and substituting the extra cysteine residues with a disulfide bridge (72–74). It has been discovered that the murine-human hybrid TCRs were able to promote engineered T cells to release cytokine and kill tumor cells compared with pure human TCR (75).

The first application of TCR-transduced T cells targeting MART-1 in patients with metastatic melanoma has been performed in 2006, and 2 of 15 treated patients showed tumor regression (76). And a clinical trial has demonstrated objective cancer regressions occur in 19 and 30% of patients with metastatic melanoma who received the gp100 or MART-1 TCR, respectively, while there was on-target toxicity presented in the eyes, ears, or skin where there was an abundance of normal melanocytes (77). Therefore, the application of TCR T-cell therapy should be careful. Unknown cross-reactive antigens may present in healthy important organs. Meanwhile, even when MAGE-A3 is not previously found in any normal organs, targeting this antigen led to 2 deaths because this TCR recognized a related epitope in MAGE-A12, which is expressed in the brain, and induced necrotizing leukoencephalopathy (78). The available strategy to avoid this reaction was the identification and evaluation of mutant-reactive TCR. A human leukocyte antigen (HLA)-C^*^08:02-restricted TCR from CD8+ TILs targeting the KRAS(G12D) hotspot driver mutation presented in many different types of human cancers such as lung, pancreatic, and gastrointestinal cancer has been identified (43).

Unfortunately, there is no clinical trial with TCR T-cell therapy for GBM in the present. But recent research has identified a TCR that targeted histone 3 variant 3 K27M (H3.3K27M) mutation that was frequently expressed in DIPG with HLA-A2 restriction. Transfer of this mutant-reactive TCR-transgenic T cells notably inhibited the progression of DIPG xenografts in mice and this provided laboratory evidence for evaluating TCR T-cell therapy targeting specific epitope in GBM, while brain inflammation during treatment should be concerned when translating into clinic (79). The general process of genetically engineered T-cell therapy (CAR- and TCR-) is shown in Figure 2B. And the existing clinical trials relating to ACT for the treatment of GBM are summarized in Table 1.

**Table 1:** Clinical trials of adoptive cell therapy in patients with glioblastoma.

**Patient number**	**Responsible party**	**Type of cells used**	**Phase**	**NCT no**.	**Status**	**Results**
14	Nabil Ahmed, Baylor College of Medicine	Anti-HER2 CAR T cells	I	NCT02442297	Recruiting	None
51	City of Hope Medical Center	Anti-HER2 CAR T cells	I	NCT03389230	Recruiting	None
16	Baylor College of Medicine	Anti-HER2 CAR T cells	I	NCT01109095	Completed	(61)
10	RenJi Hospital	Anti-EGFR CAR T cells	I	NCT02331693	Recruiting	None
60	Fuda Cancer Hospital, Guangzhou	Anti-GD2 CAR T cells	I, II	NCT03252171	Completed	None
60	Fuda Cancer Hospital, Guangzhou	Anti-EphA2 CAR Tcells	I, II	NCT02575261	Completed	None
50	Qingtang Lin, Xuanwu Hospital, Beijing	Anti- Her-2, EGFRVIII, IL13Rα2, EphA2, GD2 CD133 CAR T cells	I	NCT03423992	Recruiting	None
107	National Cancer Institute	Anti-EGFRvIII CAR T cells	I, II	NCT01454596	Completed	(65)
3	Duke University	Anti-EGFRvIII CAR T cells	I	NCT02664363	Active, not recruiting	None
24	Duke University	Anti-EGFRvIII CAR T cells	I	NCT03283631	Recruiting	None
20	Beijing Sanbo Brain Hospital	Anti-EGFRvIII CAR T cells	I	NCT02844062	Recruiting	None
92	City of Hope Medical Center	Anti- IL13Rα2 CAR T cells	I	NCT02208362	Recruiting	(66)
20	Beijing Sanbo Brain Hospital	Anti-PD-L1 CSR T cells	I	NCT02937844	Recruiting	None
332	National Cancer Institute	TILs	II	NCT01174121	Recruiting	None
10	University of Colorado, Denver	TILs	I	NCT00002572	Completed	None
83	Hoag Cancer Center, Newport Beach	LAK	II	NCT00331526	Completed	(46)

*Data from: ClinicalTrial.gov 09/10/2019*.

## Challenges

ACT depends on the identification of common and specific tumor antigens and has already exhibited prominent anti-tumor activity in cancer patients. The ACT has been successful in the treatment of hematologic malignancies and certain solid cancers such as melanoma, lung cancer, and breast cancer. However, there remain some critical challenges for the application of ACT in GBM.

### Recruitment of Infused T Cells Into GBM

Immune cells can be prevented from entering the brain parenchyma via the blood-brain barrier (BBB) under physiological conditions. Only little activated T cells are allowed to reach the brain parenchyma (80, 81). BBB consists of an endothelial basement membrane and a parenchymal basement membrane (81). Unique endothelial cells connected through tight junctions are localized on the endothelial basement membrane, where some embedded pericytes are able to found. Nevertheless, the parenchymal basement membrane is formed via astrocytic end-feet, which is also called glia limitans perivascular.

**Figure 3:**
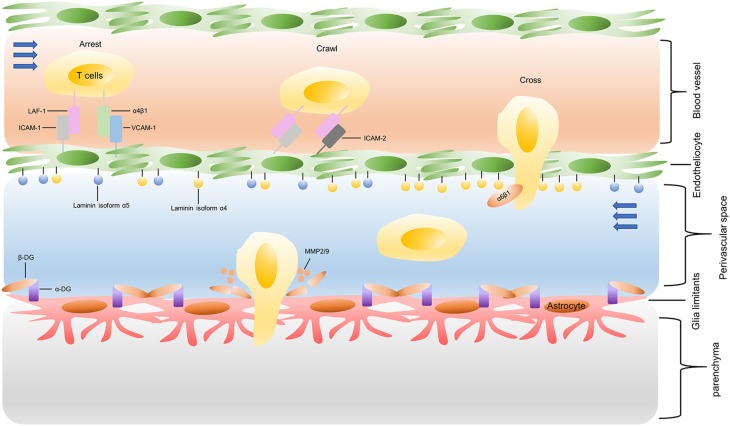
Recruitment of infused T cells into GBM. LAF-1, lymphocyte-associated antigen-1; ICAM-1, intracellular cell adhesion molecule 1; VCAM1, vascular cell adhesion molecule 1; MMP: matrix metalloproteinases; β-DG: β-dystroglycan.

The process of T cells crossing the inflamed BBB is coordinating, sequential, and complicated, as shown in Figure 3. Briefly, activated T cells initially arresting on the endothelium is mediated by the lymphocyte-associated antigen-1 (LFA-1) and α4β1-integrin expressed on the T cells, respectively binding to the intracellular cell adhesion molecule 1 (ICAM1) and adhesion molecules vascular cell adhesion molecule 1 (VCAM1) on brain endothelial cells (82). Subsequently, the T cell crawling and polarization exclusively involve LFA-1 and ICAM1/2 interactions (83). After arriving at sites where are rich in the laminin isoform α4 but not laminin α5, the T cells use α6β1-integrin to traverse the endothelial basement membrane (81). Matrix metalloproteinases (MMP)-2 and MMP-9 existing in the perivascular space promote T cells to finally cross the glia limitans into the brain parenchyma, which cleave the β-dystroglycan (β-DG, an e extracellular matrix receptor) expressed on the astrocyte end-feed (81).

Fortunately, contrast-enhanced MRI of GBM has confirmed BBB was disrupted by these tumors, while BBB is complete in the position where GBM infiltrates into the normal brain parenchyma (84). All of the above illustrate that infused T cells can arrive at GBM, while these T cells need to be activated by APCs and the efficiency of the recruitment is unclear. The route of T cell administration may affect the recruitment of infused T cells into GBM. Safety intracavitary or intratumoral delivery of T cells has been established by several investigators in clinical trials to alleviate systemic toxicity and increase T-cell homing (45, 66). Nevertheless, the intratumoral infusion has technical limitations, especially in the multi-nodulose or deep regional GBM. Additionally, intracavitary delivery of T cells does not equate to effective recruitment into the GBM and transferred T cells still must cross the BBB (85). Importantly, it is indistinct for the persistence of infused T cells about these local injections. The approach of intravenous injection promotes the persistence of transferred T cells (61, 65). A phase 1 clinical trial has shown CAR-modified T cells still exist in the peripheral blood 1 year after the infusion (61). However, it has been found that adoptive T cells initially migrate into irrelevant organs like spleen, lung, and liver, without preferential migration in tumor tissues after intravenous injection (86). Therefore, it is necessary to develop novel strategies assisting the efficient infiltration of infused T cells.

Preferential migration of T cells to tumor tissues can be induced by tumor-associated chemoattractants such as CXCL 9, 10, 11, and CCL2. Adoptive T cells can be transduced with the chemokine receptor for this chemoattractant to promote the preferential migration (87–89). However, these chemoattractants could be inhibited in highly vascular tumors such as GBM due to its capability of overproducing vascular endothelial growth factor (VEGF) (90, 91). Due to CCL7 and CCL22 that attract T regulatory cells are overexpressed in GBM, the transgene of CCR4 into adoptive T cells may improve the infiltration (92, 93). In addition, cancer endothelium overexpresses activated leukocyte cell adhesion molecule (ALCAM) in GBM. And a research group has created an ALCAM-restricted homing system (HS) to support brain cancers capture T cells (94). On the other hand, strategies designed to destroy the BBB need to be tested in the ACT, such as Focused Ultrasound (FUS), HAV6 peptide, and glutamate (95–97).

### Identification of GBM-Specific Antigens, TILs, and TCRs

**Figure 4:**
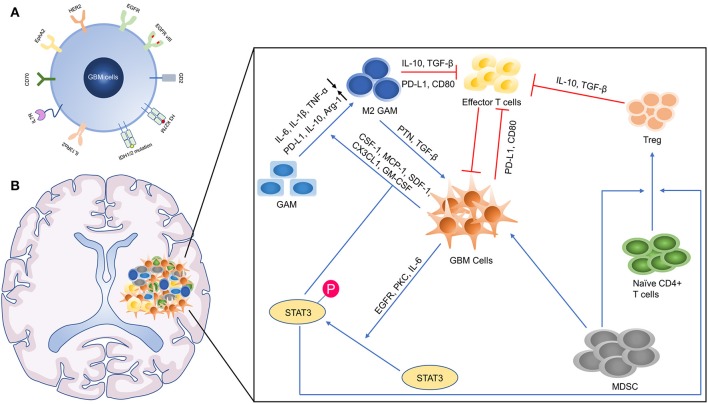
The microenvironmental landscape of GBM. **(A)** The GBM associated antigens. HER2, Human epidermal growth factor receptor 2; EGFR, epidermal growth factor receptor; GD2, disialoganglioside 2; H3 K27M, mutated histone H3 K27M; IDH, isocitrate dehydrogenase; IL13Rα2, interleukin-13 receptor alpha 2; EphA2, ephrin-A2. **(B)** The Immunosuppressive GBM microenvironment. TNF-α, tumor necrosis factor α; PD-1, programmed death 1; TGF-β, transforming growth factor-β; Arg-1, arginase 1; GM-CSF, granulocyte-macrophage colony-stimulating factor; MCP-1, monocyte chemoattractant protein 1; SDF-1, stromal cell-derived factor 1; PKC, protein kinase C.

For designing CAR-T cells, it is critical to select antigens shared with nonessential normal organs or those specific for GBM. IL13Rα2, HER2, EphA2, and EGFR have been identified as targets for GBM (Figure 4A). Although clinical trials have shown there are no severe adverse events after the administration of above CAR-T therapies, these markers may be expressed in vital organs such as lung and heart (61, 66). To overcome this potential risk, mutational antigens can be a good option, but the GBM as a type of “cold cancer” lacks pervasive mutations and it is difficult to design a CAR targeting mutations with high affinity. Importantly, some critical mutations such as isocitrate dehydrogenase (IDH) 1 (R132C/G) and BRAF (V600E) located in the cytoplasm are unable to be designed as a target for CAR-T cell therapy, which is able to be recognized by TCRs. Exploring these TCRs restricted by common HLA can be the focus of future research. Of cause, driven mutations in GBM should be detected. On the other hand, directly sorting and amplifying tumor-reactive TILs facilitate the process of ACT. Despite CD39 has been proposed as a tumor-reactive marker in CD8^+^ TILs, it needs to be further confirmed in GBM. And other markers that indicate tumor reactivity in TILs also need to be discovered. Nevertheless, the lack of tumor-reactive TILs in GBM hinders the application of TILs transfer and identification of tumor-specific TCRs. This may be resolved by combining it with dendritic vaccine therapy that can stimulate the host to produce tumor-reactive T cells (98, 99).

### Immunosuppressive GBM Microenvironment

The GBM microenvironment is developed to prevent the cancer cells from the immune attack and promote the growth of them (Figure 4B). Macrophages form the largest part of the GBM infiltrated immune cells (100). Differing remarkably from other types of cancer, macrophages possess of vary origin, including bone marrow-derived monocytes/macrophages (BMDMs) (85%) and microglia (15%). Microglia is prominent in peritumoral regions, while BMDMs recruited from peripheral blood are gathered in perivascular areas. RNA-sequencing display that BMDMs in GBM highly express genes associated with “cellular migration,” while microglia upregulate genes related to “pro-inflammatory cytokines,” which show these two subsets assume different function (101). Similarly, BMDMs and microglia are close to M2 phenotypes, producing anti-inflammatory cytokines and lacking expression of CD40, CD86, and CD80 (T cell co-stimulated molecules) (102, 103). Considering that macrophages are essential accomplices in immunosuppress, it is possible to develop effective therapies to inhibit or switch them. The administration of colony-stimulating factor 1 receptor (CSF-1R) inhibitors aimed at this goal needs to be seen, despite around 50% of animals showed resistance to these inhibitors after treatment (104). The failure of CSF-1R in the treatment of recurrent GBM patients suggests us to pay attention to the heterogeneity of macrophage (105).

In addition, another major subset of immunosuppressive cells is T regulatory (Treg) cells. Circulating T helper cells were decreased in GBM patients, whereas Treg cells were increased in the proportion of Th cells and prominently infiltrate into GBM tissues (106). In a preclinical model, the systemic application of anti-CD25 hinders Treg cell's function but not results in absolute elimination, which promotes the immune response (107). However, oxidative stress-induced apoptotic Treg cells maintain even amplify their immunosuppressive ability, explaining that the sole utilization of anti-CD25 maybe not enough (108). Moreover, immune checkpoint molecules such as CTLA-4 and PD-1 are upregulated as a result of T cell activation, which also occurs in adoptive T cells (109, 110). The combination of ACT and immune checkpoint inhibitors may promote the survival of these T cells, and adoptive T cells can be transduced with a PD-1-CD28 switch receptor that can overcome the inhibitory signaling by checkpoint activation (111).

Furthermore, a mass of abnormal molecular signaling activation involved in immunosuppression occurs in GBM, especially the activator of transcription 3 (STAT3) protein signaling. The phosphorylation of STAT3 at Ser-727 or Tyr-705 has been discovered in up to 90% GBM tissues, relating to the high histopathological grade and worse prognosis (112–114). This activation of STAT3 may not be induced by its intrinsic mutations but by upstream signaling molecules such as EGFR mutations, overexpression of interleukin 6 (IL-6) and protein kinase C (PKC) (115–117). There are certain mechanisms by which STAT3 activation can induce immune tolerance: (i) by increase of M2 like macrophages and microglia (118); (ii) through the recruitment and accumulation of Treg cells (119, 120); and (iii) via the suppression of DCs' maturation (121). The administration of WP1066 (a molecule inhibitor of p-STAT3) reverses immunosuppression in GBM patients and a clinical trial of phase I is on the way (NCT01904123) (122).

### Immune-Related Complications Associated With ACT

The ACT is correlated with life-threatening side effects, notably immune effector cell-associated neurotoxicity syndrome (ICANS) and cytokine release syndrome (CRS), which require urgent attention (123). CRS is a systemic and excessive inflammatory response initially caused by adoptive T cells through generating and releasing proinflammatory cytokines (124). These cytokines later activate endotheliocytes and bystander immune cells, formatting the vicious circle of a cytokine storm in a waterfall manner. IL-6 primarily released by macrophages seems to play a central role in CRS (125). Additionally, other cytokines like granulocyte-macrophage colony-stimulating factor (GM-CSF), monocyte chemoattractant protein 1 (MCP-1), tumor necrosis factor (TNF), interferon (IFN)-γ, and IL-1,2,8,10 participate in CRS (124).

Clinically, the symptoms of CRS may be light and limiting, or serious that require vasopressor or ventilator support. Fever is a symbolic symptom, occurring in the early phase with other mild discomforts such as myalgia, rash, headache, and arthralgia (123). Seriously, symptoms will progress to vascular leakage, hypotension, disseminated intravascular coagulation (DIC), and even multiple organ failure (MOF).

The severity of CRS is divided into 4 grades according to the American Society for Transplantation and Cellular Therapy (ASTCT) consensus guidelines (126). CRS with grade 1 is defined as a patient undergoes fevers only. Hypotension without the utilization of vasopressors and/or hypoxia with the administration of low flow oxygen occurs in grade 2 CRS patients. Grade 3 CRS requires the existence of hypotension requiring the support of one vasopressor with/without the administration of vasopressin and/or hypoxia with the use of high flow oxygen. Grade 4 CRS entails the presence of hypotension that needs multiple vasopressors excepting vasopressin and hypoxia that needs positive pressure ventilation systems, representing a life-threatening complication. It is critical to exclude infections and sepsis for the diagnosis of CRS, notably bacterial infections, and the identification of elements predicting severe CRS is developing (124, 127). Tocilizumab (an antibody against humanized IL-6 receptor) that is recommended with a maximum of 800 mg per dose and a maximum of 3 doses in 24 h and corticosteroids are the core of CRS therapy (123).

ICANS is another one of the most frequent complications in patients treated with ACT. Presently, the pathophysiology of ICANS is barely understood, while it is demonstrated that the severity of ICANS may be aggravated by a severe CRS or a high cancer burden (128). In addition, the activation of endotheliocytes and the disruption of BBB possibly lead to ICANS (129). High cytokines' levels such as GM-CSF, IL-6 present in the cerebrospinal fluid (CSF) of patients treated with ACT, resulting in brain inflammation (128). The symptoms of ICANS are more diverse than CRS, including headache, dysgraphia, aphasia, tremor, lethargy, impaired attention, and apraxia. Relatively, expressive aphasia is possibly one of the most specific symptoms in patients with ICANS (128, 130).

Similarly, the symptoms of ICANS are divided into 4 grades as well by ASTCT depending on Immune Effector Cell-Associated Encephalopathy (ICE) score (126). The preferred therapy for patients with ICANS is corticosteroids relying on the ICANS Consensus Grading. The recommended dose of dexamethasone is 10 mg per 6 h until clinical cure. However, patients with grade 4 ICANS require 1,000 mg methylprednisolone per 24 h (123, 128). Additionally, tocilizumab is absolutely recommended for patients with concurrent CRS (128).

Fortunately, present clinical trials confirm that there is no severe side-effect during the administration of ACT for GBM patients (44, 45, 61, 65, 66). It is a consensus that lymphodepletion is required before the begin of ACT by using fludarabine and cyclophosphamide to promote the persistence of infused T cells. Nevertheless, TMZ is a portion of the present therapeutic strategy for patients with GBM, which can also induce lymphopenia (131). Therefore, TMZ is supposed to be used as an inducer of lymphodepleting replacing fludarabine and cyclophosphamide (132). The pretreatment of dose-intensified TMZ induces durable lymphodepleting, whereas standard dose was transient (132). This replacement reduces the drug burden for patients who receive ACT, which entails cautious trails to interpret the dose. In addition, a high dose of IL-2 is needed for the persistence of T cells. However, the administration of IL-2 appears to lead to discomfort and even patients cannot tolerate it. It has been revealed that adoptive T cells engineered to express IL-12 were able to survive in the absence of IL-2 while they cannot stay long-term in the host (133).

## Conclusion

To conclude, ACT is a highly personalized therapy that possesses a significant potential for the treatment of different types of cancers. GBM is unique in terms of its characteristics like low immunogenicity, immunosuppression, and exclusive location, all of which make it particularly difficult to treat. ACT can be a potential therapy for the treatment of GBM. Further researches are necessary to solve the above challenges and a combination of several immunotherapies may prove to be a solution.

## Author Contributions

All authors participated in the preparation of this manuscript. JH and YZ were responsible for determining the topic. YZ, JW, FS, and YY collected data. JW, FS, and JH contributed to writing the first draft of this article. LW was responsible for furtherly editing the manuscript.

### Conflict of Interest

The authors declare that the research was conducted in the absence of any commercial or financial relationships that could be construed as a potential conflict of interest.

## Abbreviations

GBM: glioblastoma multiforme
ACT: adoptive cell therapy
TIL: tumor-infiltrating lymphocyte
CNS: central nervous system
TTF: tumor-treating fields
mAB: monoclonal antibodies
CTLA4: cytotoxic T lymphocyte-4
PD-1: programmed death 1
OVs: oncolytic viruses
DCs: dendritic cells
CAR: chimeric antigen receptor
RT: radiotherapy
TMZ: temozolomide
HR: hormone receptor
PBMC: peripheral blood mononuclear cell
TM: transmembrane
TAA: tumor-associated antigen
MHC: major histocompatibility complex
scFv: single-chain variable fragment
EGFR: epidermal growth factor receptor
IL13Rα2: interleukin-13 receptor alpha 2
HER2: human epidermal growth factor receptor 2
EphA2: ephrin-A2
PDPN: podoplanin
DIPG: diffuse intrinsic pontine glioma
H3-K27M: mutated histone H3 K27M
GD2: disialoganglioside 2
TCR: T cell receptor
HLA: human leukocyte antigen
VEGF: vascular endothelial growth factor
BBB: blood-brain barrier
LFA-1: lymphocyte- associated antigen-1
ICAM1: intracellular cell adhesion molecule 1
VCAM1: vascular cell adhesion molecule 1
MMP: matrix metalloproteinase
β-DG: β-dystroglycan
ALCAM: activated leukocyte cell adhesion molecule
HS: homing system
FUS: Focused Ultrasound
IDH: isocitrate dehydrogenase
CSF-1R: colony stimulating factor 1 receptor
STAT3: activator of transcription 3
PKC: protein kinase C
ICANS: immune effector cell-associated neurotoxicity syndrome
CRS: cytokine release syndrome
GM-CSF: granulocyte-macrophage colony-stimulating factor
MCP-1: monocyte chemoattractant protein 1
TNF: tumor necrosis factor
DIC: disseminated intravascular coagulation
MOF: multiple organ failure
ASTCT: American Society for Transplantation and Cellular Therapy
CSF: cerebrospinal fluid
TGF-β: transforming growth factor-β
IL-10: interleukin 10
TAMs: tumor-associated macrophages
MDSCs: myeloid-derived suppressor cells.
